# Disrupting the Repeat Domain of Premelanosome Protein (PMEL) Produces Dysamyloidosis and Dystrophic Ocular Pigment Reflective of Pigmentary Glaucoma

**DOI:** 10.3390/ijms241914423

**Published:** 2023-09-22

**Authors:** Elizabeth D. Hodges, Paul W. Chrystal, Tim Footz, Lance P. Doucette, Nicole C. L. Noel, Zixuan Li, Michael A. Walter, W. Ted Allison

**Affiliations:** 1 Department of Biological Sciences, University of Alberta, Edmonton, AB T6G 2E9, Canada; edhodges@ualberta.ca (E.D.H.); pchrysta@ualberta.ca (P.W.C.);; 2 Faculty of Science, University of Alberta, Edmonton, AB T6G 2E9, Canada; 3 Department of Cell & Systems Biology, University of Toronto, Toronto, ON M5S 3G5, Canada; 4 Department of Medical Genetics, University of Alberta, Edmonton, AB T6G 2R3, Canadamwalter@ualberta.ca (M.A.W.); 5 Institute of Ophthalmology, University College London, London EC1V 9EL, UK; 6 Centre for Prions & Protein Folding Disease, University of Alberta, Edmonton, AB T6G 2M8, Canada

**Keywords:** melanin, melanosome, prion-like, SILV, PMEL17, retina, pigment dispersion syndrome

## Abstract

Pigmentary glaucoma has recently been associated with missense mutations in *PMEL* that are dominantly inherited and enriched in the protein’s fascinating repeat domain. PMEL pathobiology is intriguing because PMEL forms *functional* amyloid in healthy eyes, and this PMEL amyloid acts to scaffold melanin deposition. This is an informative contradistinction to prominent neurodegenerative diseases where amyloid formation is neurotoxic and mutations cause a toxic gain of function called “amyloidosis”. Preclinical animal models have failed to model this PMEL “dysamyloidosis” pathomechanism and instead cause recessively inherited ocular pigment defects via PMEL loss of function; they have not addressed the consequences of disrupting PMEL’s repetitive region. Here, we use CRISPR to engineer a small in-frame mutation in the zebrafish homolog of *PMEL* that is predicted to subtly disrupt the protein’s repetitive region. Homozygous mutant larvae displayed pigmentation phenotypes and altered eye morphogenesis similar to presumptive null larvae. Heterozygous mutants had disrupted eye morphogenesis and disrupted pigment deposition in their retinal melanosomes. The deficits in the pigment deposition of these young adult fish were not accompanied by any detectable glaucomatous changes in intraocular pressure or retinal morphology. Overall, the data provide important in vivo validation that subtle *PMEL* mutations can cause a dominantly inherited pigment pathology that aligns with the inheritance of pigmentary glaucoma patient pedigrees. These in vivo observations help to resolve controversy regarding the necessity of PMEL’s repeat domain in pigmentation. The data foster an ongoing interest in an antithetical dysamyloidosis mechanism that, akin to the amyloidosis of devastating dementias, manifests as a slow progressive neurodegenerative disease.

## 1. Introduction

Pigmentary glaucoma is a complex form of vision loss with only a single known causative gene, *PMEL* (encoding the premelanosome protein), which was identified by a recent international collaboration considering patients from multiple countries and lineages [1]. The PMEL protein is processed into an abundant functional amyloid that forms the fibrillar scaffold for the deposition of eumelanin pigment and elongates the melanosome (the pigment-containing organelle) during melanosome development [2,3,4,5,6]. The disease mechanism associated with these mutations is unknown, limiting successful medical management. 

Well-documented examples of *functional* amyloid are very rare in vertebrates [7,8,9,10,11,12]. Opportunities to dissect the cell biology of amyloid homeostasis, and determine how cells thrive with abundant amyloid, are of substantial interest: the approach provides a distinct and insightful perspective on how therapeutics might ameliorate damage from the mangles of amyloid that produce devastating dementias, including Alzheimer’s and Parkinson’s diseases. Recently identified human mutations in the amyloidogenic components of PMEL [1] provided additional inspiration to consider how (or if) subtle alterations in a functional amyloid can manifest as a “dysamyloidosis” and a slow progressive neurodegenerative disease, i.e., a heritable form of pigmentary glaucoma. 

PMEL’s repeat domain is thought to be integral to amyloid formation, and similar repetitive regions are central to other (neurotoxic) amyloidogenic proteins [7,8,9,10,11,12]. This has been debated, however, because in vitro work on this region of human, mouse and zebrafish PMEL have shown that it is sufficient or dispensable for amyloid fibril formation and pigment deposition [8,13,14,15,16]. This debate, alongside the potential for a dysamyloidosis aetiology, prompted our investigation of whether PMEL’s repeat domain is necessary in vivo. 

**Figure 1:**
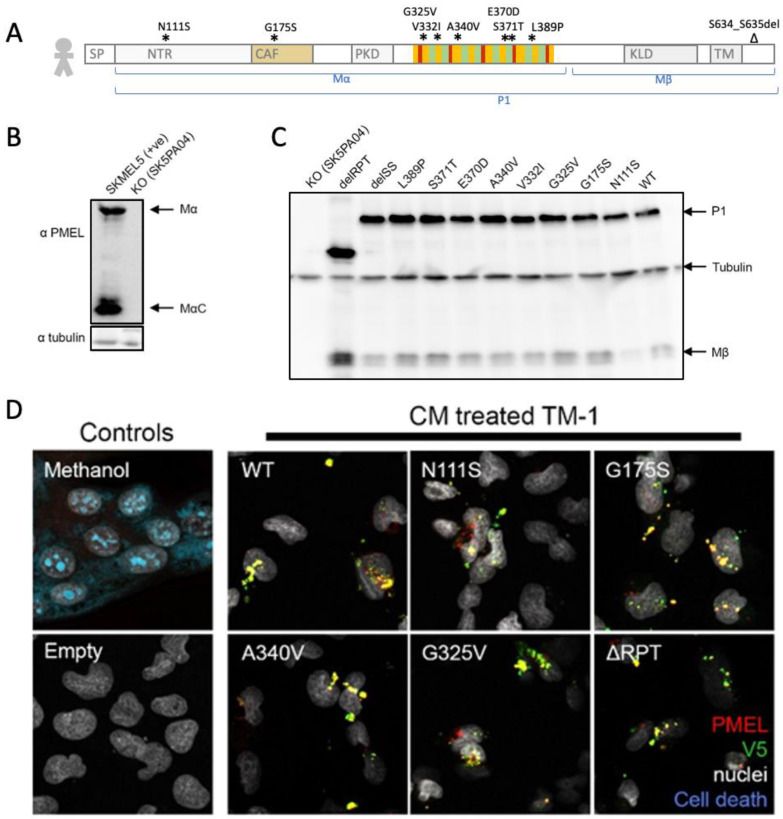
Human PMEL is engulfed by trabecular meshwork cells, providing a *potential* route for pigment cell defects to influence ocular drainage and glaucomatous increases in intraocular pressure. However, our surface-level phenotyping of TM-1 cell health did not reveal any overt toxicity to the cells engulfing patient variant PMEL; further work is warranted before any confident interpretations about the lack of toxicity or functional disruption can be concluded. (**A**) Schematic of human PMEL protein (in sync with Figure 2A), detailing the identity of pigmentary glaucoma patient variants. Details of the repeat region are simplified/omitted here, but they are presented in Figure S1. PMEL processing via cleavages produces bands Mα, Mβ and P1, with the latter two being detectable by a C-terminal V5 tag. Symbols * and ∆ indicate positions of pigmentary glaucoma patient variants, including an enrichment of missense mutations in the repeat domain. (**B**) SKMEL5 cells were edited with CRISPR to knockout (KO) endogenous PMEL, generating clone SK5PA04 that showed a lack of PMEL when immunoblotted with anti-PMEL antibody HMB45. (**C**) Patient variant PMEL with V5 tag was transfected into SK5PA04 cells and blotted with V5 antibody. (**D**) Trabecular meshwork cells (TM-1) engulfed PMEL (V5-tagged) supplied by conditioned media from transfected SK5PA04 cells; notice the lack of signal in TM-1 cells incubated with conditioned media from SK5PA04 cells with empty vector. Engulfment of patient variant PMEL did not lead to a detectable reduction in cell viability, but further work would be needed to assess long-term health and function of these TM cells, perhaps including the use of in vivo platforms.

Pigmentary glaucoma patients exhibit a dominant inheritance, and these mutations are enriched in the PMEL repeat domain (Figure 1A) [1]. In a recent review, we collated PMEL mutations from patients, from preclinical animal models and from diverse domesticated animal species. This demonstrated a consistent pattern where nonsense and null mutations exhibit recessive inheritance (of pigment and/or ocular defects), whereas missense *PMEL* mutations exhibit dominant inheritance [17]. The dominant missense mutations in *PMEL*, including in pigmentary glaucoma patients, suggest the hypothesis that mutant PMEL amyloid in heterozygous animals has intermolecular interactions that disrupt the function of the normal PMEL protein. An alternative explanation is haploinsufficiency, though this is not supported by the observation that heterozygous carriers of null mutations do not exhibit overt phenotypes. Regardless, dominant inheritance of PMEL mutant phenotypes has not been observed in any preclinical animal models (mice and zebrafish) to date, and only null mutations (or strong hypomorphs or knockdowns) in PMEL have been examined in regards to pigmentation disruption (Silv mutant mice [18], and zebrafish mutants and morphants [1,19,20]). Thus, pigmentary glaucoma patients exhibit dominant missense mutations (and these are predominantly located in the repeat domain), yet all available preclinical models (e.g., mice or zebrafish) are nonsense mutations with recessive inheritance. The work presented here begins to fill this gap. 

The precise pathologic pathway resulting in pigmentary glaucoma remains unknown in humans. Pigmentary glaucoma is often preceded by pigment dispersion syndrome wherein pigment is sloughed from the posterior side of the iris and freely follows the flow of the aqueous humour [21]. Hypotheses of why the pigment is released into the anterior chamber include either a cytotoxic effect of PMEL and/or a role for anterior segment conformational abnormalities that may cause mechanical damage to the iris through the rubbing of zonular fibres [21]. The pigment could increase eye pressure (a hallmark of and risk factor for glaucoma) either by physically blocking and/or damaging the trabecular meshwork cells that control the drainage of aqueous humour out of the eye [22].

Either of these potential mechanisms would increase intraocular pressure and the risk of glaucoma/optic nerve death and, ultimately, vision loss. Additional hypotheses consider the cytotoxic or developmental/anatomical effects of PMEL that are independent of ocular hypertension; these warrant consideration because not all pigmentary glaucoma patients have increased eye pressure, and the full extent of the roles of the PMEL protein in ocular development remain unknown [21]. However, elevated intraocular pressure does remain a prominent risk factor for and symptom of glaucoma, in addition to being a common treatment target.

We report on a new zebrafish *pmela* mutant with an in-frame deletion that is predicted to subtly disrupt the protein’s repetitive region (designated as the *ua5030* allele, a twelve-base-pair deletion). We characterize these mutants with biological assays and complement this with clinically inspired ophthalmological evaluations via optical coherence tomography and rebound tonometry.

## 2. Results

### 2.1. Patient PMEL Variants Supplied in Supernatant Are Engulfed by Trabecular Meshwork Cells In Vitro but Show No Measurable Toxicity

Considering the proposed mechanisms of pigmentary glaucoma (see Section 1), its dominant inheritance in patients and the propensity of amyloid-related disease to spread in a prion-like fashion, we speculated that patient mutations in *PMEL* could act in a non-cell-autonomous fashion to disrupt cells in the trabecular meshwork (TM, thereby disrupting ocular drainage and creating glaucomatous increases in intraocular pressure). SKMEL5 cells were edited with CRISPR to remove the endogenous expression of PMEL (Figure 1B), providing a melanosome-friendly platform for the expression of human PMEL protein variants. V5-tagged PMEL representing the patient variants (detailed in Figure 1A) or the wildtype was expressed efficiently when transfected into these SKMEL^PMEL-KO^ cells (Figure 1C).

Intriguingly, PMEL appears to be readily engulfed by TM cells, which was apparent when the conditioned media from the transfected SKMEL5 cells were applied to TM-1 cells (Figure 1D; notice that the signal is absent when the media were sourced from empty vector negative control SKMEL5 cells lacking PMEL). Compared to wildtype, the PMEL patient variants engulfed by TM-1 cells did not display overt differences in PMEL distribution or any overt elevations in cell death (Figure 1D). Further work would be required before any claims could be made from these data regarding patient variant PMEL and its potential impacts on TM health and function. This includes, but is not limited to, assessing cells after longer durations of PMEL incubation, assessing PMEL patient variants and quantifying various metrics of cell viability. However, the absence of overt and tractable PMEL defects led us to consider the potential limitations of in vitro systems and instead focus our efforts on in vivo modelling. Regardless, this work strongly supports the contention that non-cell-autonomous uptake of mutant PMEL can occur in the TM cells that support ocular drainage, and, thus, this remains a hypothetical mechanism for PMEL variants to alter intraocular pressure. This speculative non-cell-autonomous pathomechanism requires only a single copy of *PMEL* to harbor amyloid mutations, consistent with a dominant inheritance pattern.

### 2.2. Zebrafish Pmela Mutant Engineered to Reflect Human Patient Variations in PMEL

**Figure 2:**
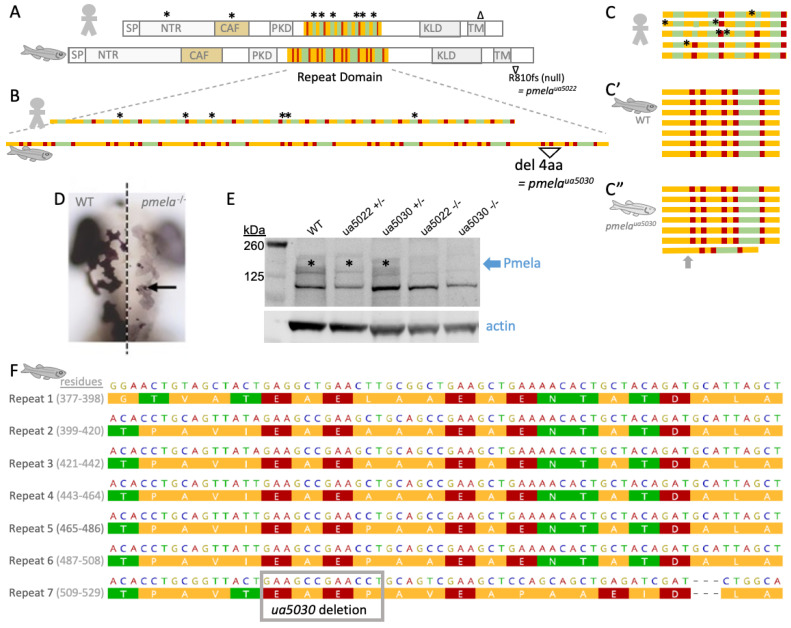
A zebrafish model of pigmentary glaucoma engineered via subtle mutation to the repeat region of the *PMEL* homolog that forms functional amyloid in pigment cells. (**A**) Homologs of PMEL protein in human and zebrafish are strikingly similar with a full complement of shared domains, including a repeat domain that contributes to making functional amyloid. Zebrafish Pmela protein is ~25% longer than human PMEL. The repeat domain in PMEL is homologous between human and zebrafish, though the details of which amino acids comprise each repeat are different; colours in panels (**A**–**C**) present a simplified schematic of amino acid properties (polarity) in these repeats, with detailed views in panel F and Figure S1. Symbols * and ∆ above human PMEL indicate positions of pigmentary glaucoma patient variants (details in Figure 1), including an enrichment of missense mutations in the repeat domain [1]. (**A**) Previously engineered zebrafish mutant *pmela^ua5022^* creates a premature stop codon in the N-terminus of zebrafish Pmela, and this is a null allele likely due in part to nonsense-mediated decay [1]. (**B**,**C**) Human vs. zebrafish repeat domains (simplified to emphasize repetitive aspects; see Figure S1 for details of exact amino acid content). Here, we engineered a zebrafish mutant *ua5030* with an in-frame 12 bp deletion leading to a predicted loss of only 4 residues (“del 4aa”). Panel (**C**) presents the same protein sequences as panel B but reconfigured to emphasize the repetitive nature of the PMEL repeat domain. Human PMEL contains 5 repeats of 26 amino acids (**C**), in comparison to zebrafish Pmela (**C’**), which has 7 repeats of 22 amino acids. Akin to the non-synonymous mutations in pigmentary glaucoma patients (*), the deletion in Pmela*^ua5030^* (**C”**) is predicted to subtly disrupt the overall protein but alter residues that show a rigidly repetitive character. (**D**) Pigment defect in larval *pmela* mutant zebrafish. Dorsal view, anterior at top, merge of wildtype (WT) and mutant larvae to allow side-by-side comparison at dorsal midline (dotted line). (**E**) Homozygous zebrafish *pmela* mutant larvae lacked detectable Pmela protein (both alleles). Pmela abundance was perhaps reduced in heterozygous *pmela*^+/ua5022^ but appeared similar to WT in heterozygous *pmela*^+/ua5030^ larvae. Custom Anti-Pmela antibody was raised against the repeat domain of zebrafish Pmela. (**F**) Zebrafish Pmela repeat region (NP_001038795.1 residues 377-529) with amino acid properties colour-coded by polarity and stacked to emphasize the rigidity of repeat identity (repeats R1 to R7 are listed alongside their residue numbering). Mutation *pmela^ua5030^* deletes 12 bp to create a 4-residue in-frame deletion. SP = signal peptide; NTR = N-terminal region; CAF = core amyloid fragment; PKD = polycystic kidney domain; KLD = Kringle-like domain; TM = transmembrane domain.

CRISPR mutagenesis was used to engineer an in-frame 12 bp deletion within the repeat domain of the zebrafish *pmela*, generating an allele designated *ua5030* (Figure 2). This mutation is predicted to leave most of the Pmela protein intact, except for deleting four residues in the C-terminal portion of the repeat domain (Figure 2B,C”,F). The efficacy of this mutation in disrupting *pmela* gene products was supported by the hypopigmented phenotype (Figure 2D) and the reduced abundance of *pmela* transcripts in the 3 dpf larvae, presumably resulting from the decay of the mutant transcript (Figure S2A). The Pmela protein was less abundant in the homozygous ua5030 mutant larvae, as determined via immunoblotting with a custom antibody raised against the repeat domain of zebrafish Pmela (Figure 2E and Figure S2). Immunoblotting revealed an appropriate product in the wildtype larvae and adult eyes, and this band was absent in the same tissues from the adult homozygous ua5022 individuals (Figure S2) that have a mutation leading to C-terminal truncation and are believed to be a null or strong hypomorph [1]. Pmela protein abundance was reduced in the *pmela*^+/ua5022^ heterozygotes, though it was less obviously reduced in the *pmela*^+/ua5030^ heterozygotes (Figure 2E). These protein abundances could be reflective of an altered transcript abundance considering that this functional amyloid protein is expected to be relatively stable [7,8,9,10,11,12].

The repeat domain is homologous in zebrafish and human PMEL, a location where two-thirds of the human pigmentary glaucoma patient mutations cluster (Figure 2A). From a bioinformatic perspective, it seems plausible that a small deletion in the repeat domain (as in *ua5030*) may be impactful on function. In particular, a rigid consistency of amino acids is apparent between the seven rungs of Pmela repeats (seven repeats of ~22 residues each; Figure 2C); this intense conservation between repeats suggests strong selection pressure and required function(s) of the repeat domain. Considering the role of this repeat domain in forming amyloid, it is probable that the rigidly consistent repeats modulate intermolecular interactions among adjacent Pmela proteins towards their fibrillization. Similar speculations can extend to the human PMEL repeat domain (Figure 1A, Figure 2B,C and Figure S1), where missense mutations cause pigmentary glaucoma and disrupt amyloid formation when expressed in HeLa cells [1]. The subtle disruption in Pmela^ua5030^ is more representative of these human patient variants compared to previously described mouse models or zebrafish mutants or morphants, which are nulls, strong hypomorphs or predicted to lack several key protein domains [5,19,20].

### 2.3. Zebrafish Pmela Mutants Are Grossly Hypopigmented

**Figure 3:**
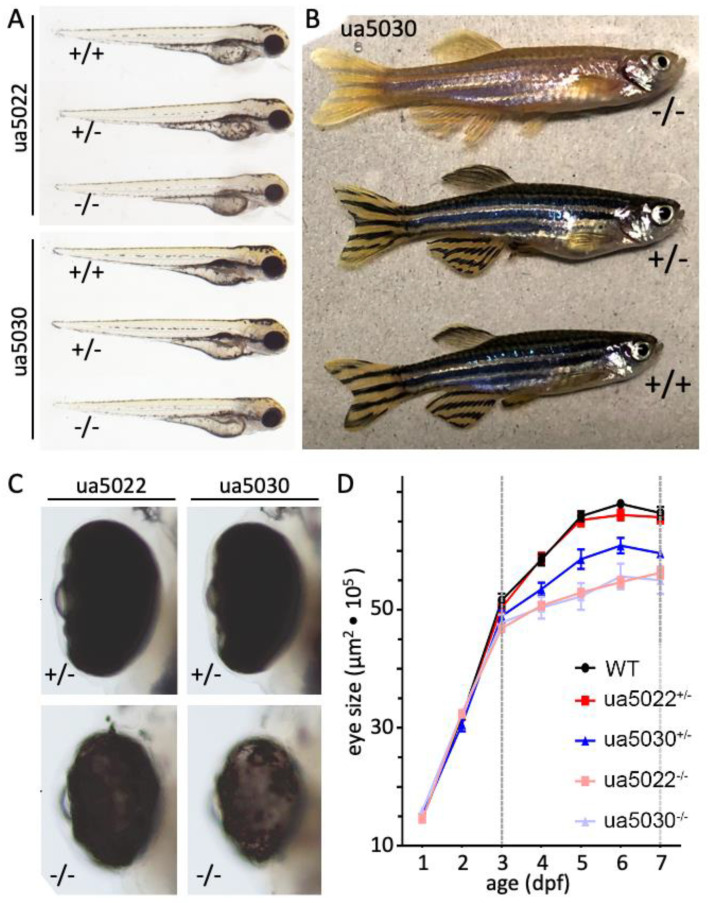
*pmela* mutants display systemic hypopigmentation and retarded eye size development. (**A**) Both *pmela* mutant alleles present with systemic hypopigmentation in homozygotes at 3 days post-fertilization (dpf). (**B**) Adult homozygous ua5030 zebrafish (−/−, top) display hypopigmentation when compared to heterozygous and wildtype fish. (**C**,**D**) Microphthalmia is observed in homozygous mutants from both alleles beginning by 4 dpf (days post-fertilization). Heterozygous *pmela*^+/ua5022^ larvae show normal eye size, whereas heterozygous *pmela*^+/ua5030^ larvae have reduced eye size. Considering the normal abundance of Pmela in *pmela*^+/ua5030^ larvae (Figure 2E), this appears to be a dominant phenotype rather than haploinsufficiency.

Both alleles of zebrafish *pmela* mutants were grossly hypopigmented when bred to homozygosity (Figure 3A–C). This was expected for the previously published null allele (*ua5022*) [1], and this is consistent with body colouration being diluted in *PMEL* mutants across several taxa [17]. The observation that that this phenotype was recapitulated in the *ua5030* mutant demonstrates that an intact repeat domain is required for normal pigmentation in vivo.

### 2.4. Larval Zebrafish Pmela Mutants and ua5030 Heterozygotes Have Microphthalmia

The *pmela* homozygote mutants from both alleles had reduced eye size starting at 3 days post-fertilization and persisting until at least 7 days (Figure 3C,D). The ua5030 heterozygote had an intermediate microphthalmia between the homozygote *pmela* mutants and wildtype larvae (Figure 3D).

### 2.5. Adult Zebrafish Pmela Mutants Do Not Have a Grossly Apparent Ocular Phenotype

**Figure 4:**
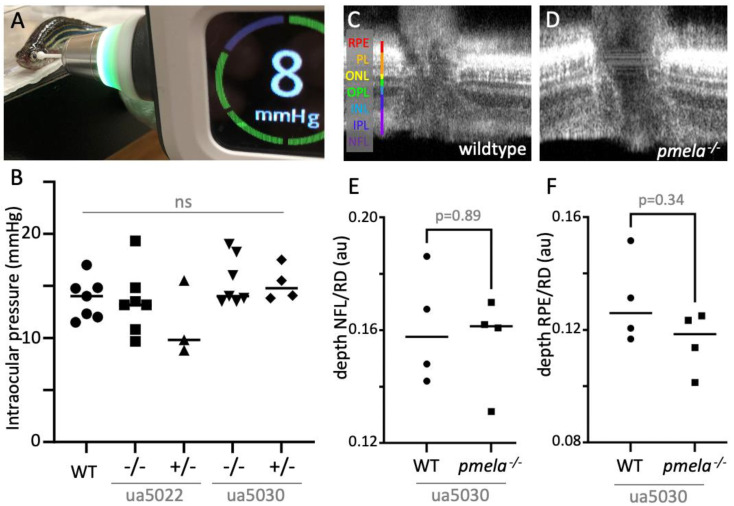
Adult *pmela*^−/−^ mutants presented no measurable phenotypes when evaluated via rebound tonometry and ocular computed tomography (OCT). (**A**,**B**) No consistent difference in intraocular pressure (IOP) was apparent among adult zebrafish of various *pmela* genotypes. IOP was measured in adult zebrafish using Tonovet plus. Average readings for each fish are plotted. A Kruskal–Wallis statistical test showed no significant differences between means. (**C**–**F**) Retinal lamination and layer depths showed no overt alterations when *pmela* mutants were assessed in vivo via OCT. Exemplar images are shown for adult wildtype and ua5030 homozygotes, whereas ua5022 homozygotes and measurements of additional layers are reported in Figures S3 and S4. The ratio of each retinal layer was normalized to the full retinal depth (RD) for each fish. RPE, retinal pigment epithelium; PL, photoreceptor layer; ONL, outer nuclear layer; INL, inner nuclear layer; IPL, inner plexiform layer; NFL, neurofibrillary layer. Comparison with age-matched controls used Mann–Whitney statistical tests. The horizontal lines in the graphs B, E and F represent the mean values of multiple fish and the value for each individual fish is plotted as a filled symbol (circle, square, rectangle, diamond, etc.).

In mature fish, no marked ocular changes were observed in the *ua5022* or *ua5030 pmela* homozygous mutant fish (Figure 4). The intraocular pressure measurement showed no detectable difference between the different genotypes (Figure 4A,B). These measures were attained with a new and more accessible method of determining intraocular pressure in zebrafish that produces results in wildtype zebrafish that are similar to those achieved via the previously described servonull methods [23].

Optical coherence tomography (OCT) was used to measure the relative depth of the retinal layers adjacent to the optic nerve and detected no difference between the *pmela* mutants and age-matched wildtype (AB) individuals (Figure 4C–F, Figures S3 and S4).

### 2.6. Pmela Mutants Have Smaller Melanosomes with More Variable Electron Density

**Figure 5:**
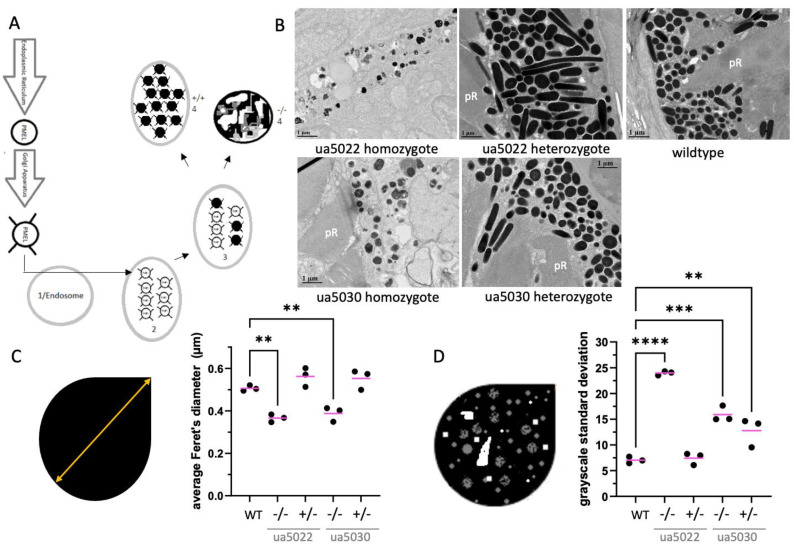
The repeat region of the Pmela protein is required for the elongation of melanosomes and the even distribution of melanin within melanosomes. (**A**) Schematic of the maturation of premelanosome protein (Pmel) and the melanosome. Pmel is made in the endoplasmic reticulum and modified in the Golgi apparatus before entering an endosome. The endosome then goes through four stages (1 → 4) of maturation before becoming a fully formed (WT) melanosome. Stage 1: fibril-free endosome; Stage 2: fibrils begin forming; Stage 3: melanin starts to be deposited on the fibrils; Stage 4: melanin is evenly distributed, obscuring the fibrils. Melanosomes are oblong in shape (WT). In *Pmel*-knockout mice (−/−), the mature melanosome has irregularly distributed melanin and is round like in Stage 1 [18]. (**B**) Sample transmission electron microscopy images of retinas from 5-day post-fertilization zebrafish of various *pmela* genotypes: wildtype, ua5022 homozygous and heterozygous, and ua5030 homozygous and heterozygous. pR = photoreceptor. (**C**) A pictograph of measuring Feret’s diameter (orange arrow—the longest distance that could be measured in a straight line within the confines of the melanosome) accompanies the results from the different genotypes; the ANOVA with Tukey’s post hoc test shows the average Feret’s diameter of melanosomes is shorter in the two homozygote mutants, ua5022 and ua5030, when compared to that of the wildtype. (**D**) Melanin deposition is uneven in several *pmela* genotypes. The average grayscale value is measured by pixels within single melanosomes, and the grayscale standard deviation is calculated on individual melanosomes. This standard deviation is used as a metric for general variation within all the melanosomes of an individual. Wildtype melanosomes are homogenous with low standard deviation; in contrast, mutants have variable electron density due to unequal distribution (clumping) within the melanosomes. An ANOVA with Tukey’s post hoc test shows the melanin deposition in melanosomes (average grayscale standard deviation) is more variable in ua5022 homozygotes, and in both ua5030 homozygotes and heterozygotes. ** *p* < 0.01, *** *p* < 0.001; **** *p* < 0.0001. See Supplemental Figure S5 for individual melanosome data.

In homozygote animals from both *pmela* alleles, the morphology of the melanosomes in the larval retinal pigmented epithelium failed to elongate, consistent with melanosomes described with other loss-of-function mutations in *PMEL* homologs (Figure 5A–C). To quantify this, we calculated the average Feret’s diameter (the longest straight line that can be measured within the melanosome) of many melanosomes from each of the three individuals of each genotype. Both the *ua5022* and *ua5030* homozygous melanosomes were roughly one-fifth shorter than wildtype (Figure 5C). These differences were significant (*p* < 0.01) when comparing between groups of fish (n = 3 fish per genotype), but the morphological difference was also apparent in the raw data (dozens of melanosomes quantified per individual, Figure S5A).

The melanosomes observed in both *pmela* mutants also exhibited an obvious variability in their electron density (Figure 5B), contrasting with wildtype melanosomes that had a homogenous electron density. To quantify this variability, we calculated the standard deviation of the grayscale values (assigned to each pixel) within each melanosome. When the average of these values was compared for each genotype (n = 3 fish per genotype), both homozygous *pmela* mutant alleles were found to have a substantially increased (more than doubled) variability in melanosome density (Figure 5D). The average values for each fish were used to ensure a conservative statistical analysis, but the considerable variation in the mutant melanosomes was also well represented by the raw data of the grayscale variation within each melanosome (dozens of melanosomes measured per individual fish, with three individual fish per genotype, Figure S5B).

Intriguingly, the melanosomes in the *ua5030* heterozygous mutants exhibited considerable variability in their electron density that was not observed in the wildtype or *ua5022* heterozygotes (Figure 5D). The mean of the density variability in the melanosomes for each individual demonstrated a doubling in the *ua5030* heterozygotes compared to in the wildtype or *ua5022* heterozygotes. The variability in density within each melanosome is provided for a visualization of the variability within each of the three individuals of each genotype (Figure S5B). Considering that the abundance of the Pmela protein was not detectably reduced in the *ua5030* heterozygotes (Figure 2, compared to the wildtype), this dramatic variability in melanin density within the heterozygous *ua5030* melanosomes (contrasting the *ua5022* heterozygote melanosomes) is best interpreted as a dominant phenotype resulting from a small perturbation of the Pmela repeat region in one copy of the *pmela* gene (see Section 3).

### 2.7. The outer Retina of Pmela Mutants Show Varied Delays and Perturbation of Development

**Figure 6:**
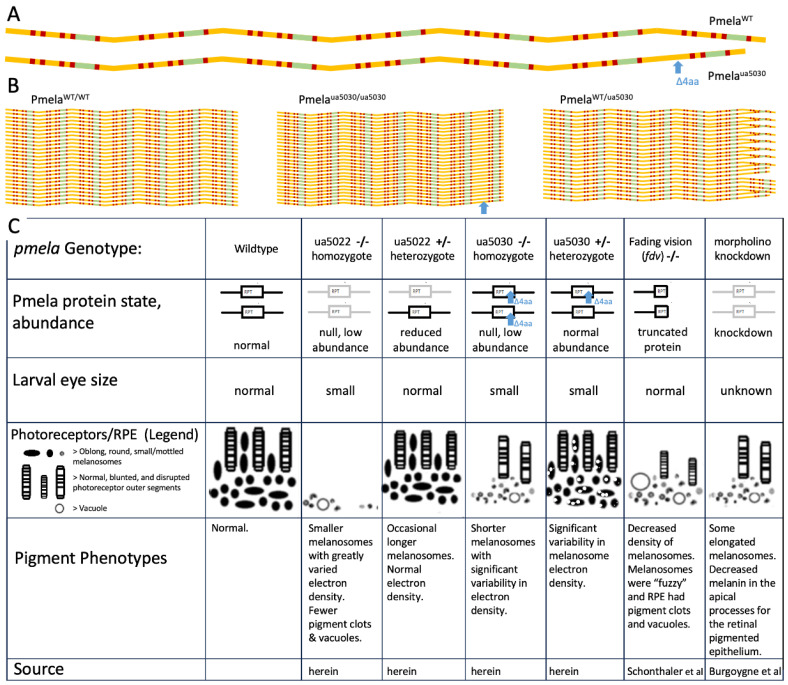
Summary of *pmela* genotype–phenotype relations and illustrations suggesting how the heterozygous *ua5030* phenotype might be attributed to aberrant fibril formation and melanin deposition. (**A**) The linear wildtype (WT) peptide has a subset of residues colour-coded for their biochemical properties, akin to Figure 2. There are seven repeated modules. We assume a small conformational change (details unknown) at the location of the 4-residue deletion (Δ4aa, blue arrow), represented here by the absence of a slight bend in the peptide shape at the C-terminal repeat. (**B**) Homozygous mutant Pmela can stack together effectively (akin to WT), where the mutant is disrupted (e.g., 4 residues shorter) consistent with lost function (abundance of this protein is low in homozygous mutants). In heterozygous Pmela^WT/ua5030^ fish, the melanosome has a mixture of two different peptides that try to assemble, and the stacking is disrupted (apparent on right side of the stack). Top half of the heterozygous stack imagines a 1:1 ratio and exactly reiterated WT/mutant/WT/mutant peptides, and bottom of stack imagines a more random recruitment of WT vs. mutant peptides. This is meant to represent how the intermolecular interaction of PMEL variants might be impactful in the heterozygous state (disrupting melanin pigment deposition and melanosome shape), consistent with a dominant inheritance. Imagine each stack is extended top and bottom to make a fibril, and fibrils then form a scaffold for both melanin deposition and melanosome morphogenesis. This simplified schematic ignores various details of PMEL biochemistry. (**C**) Summary of how pigmentation phenotypes differ in various disruptions. Repeat domain (RPT) presented as a box, protein schematic summarizes the different phenotypes using gray colouration for hypomorphs or null mutations, and truncations in fdv are schematized shorter. The legend shows the various changes to the photoreceptors and retinal pigmented epithelium: see text and previously published works [1,19,20]. Near-absence of Pmela or loss-of-function mutation produces pigment phenotypes, but moderately reduced abundance of Pmela does not. Phenotypes in ua5030 heterozygotes are consistent with an altered RPT domain leading to dominant inheritance of pigmentation deficits.

Ocular sections of 5dpf zebrafish retinas, evaluated using transmission electron microscopy, are summarized in Figure 6 and were compared to those in the pre-existing literature for each genotype. The homozygous *ua5022* larvae had no to very few photoreceptors (Figure 5B). This was also the case in two-thirds of the *ua5030* homozygotes, with the remaining individual having photoreceptors with abnormal outer segments. The homozygote mutant retinal pigmented epithelium had “pigment clots” (aggregations of abnormal pigmented organelles in the retinal pigmented epithelium), many vacuoles and a decreased number of melanosomes between individuals and across each retina (Figure 5B and Figure 6). Neither of the heterozygote larvae showed these same effects.

## 3. Discussion

### 3.1. The ua5030 Phenotype Highlights the Importance of the Repeat Region of Pmela

The pronounced novel heterozygous *pmela* phenotype, resulting from the small in-frame mutation (*ua5030*) in the repeat region of Pmela, supports the importance of the region (Figure 2, Figure 3 and Figure 5). Zebrafish and humans are the only animals known to have phenotypes from mutations in this domain, and it is remarkable that, in zebrafish, affecting only the repeat domain caused similar changes to those described in previously published null mutations (*ua5022*, *fading vision*; [17,20]). Considering the presumed role of the repetitive region in amyloid fibril formation, fibril derangement or some other toxic effect underpinning Pmela’s altered functioning is a natural prediction [8,13,14,15,16]. The mutation could impact not only the conformation and function of the individual Pmela protein molecules but also how they interact with the other molecules of the Pmela protein (Figure 6A,B).

One hypothesis to explain these data is that small disruptions to the Pmela repeat domain can cause dominant inheritance, consistent with human pedigrees. That hypothesis is supported by the heterozygous repeat domain mutant (*pmela*^ua5030/+^) larvae displaying two phenotypes: small eye size (Figure 3D) and uneven pigment deposition within melanosomes (Figure 5D and Figure S5B). An alternative explanation for these phenotypes in the *pmela*^ua5030/+^ zebrafish is haploinsufficiency. Two lines of evidence make haploinsufficiency an unlikely explanation. First, the abundance of the Pmela protein was not dramatically reduced in the *pmela*^ua5030/+^ larvae, whereas it appeared to be reduced in the *pmela*^ua5022/+^ larvae (Figure 2E). More incisively, these phenotypes both appeared only in the heterozygous *pmela*^ua5030/+^ larvae and not in the heterozygous *pmela*^ua5022/+^ larvae (summarized in Figure 6C), despite the latter genotypes having similar or greater reductions in the Pmela protein. Moreover, homozygous *pmela*^fdv/fdv^ mutants show very similar pigmentation defects to what we describe here, with recessive inheritance and no such defects reported for heterozygous *pmela*^fdv/+^ mutants [20]. Thus, the ua5030 allele appears distinct from past *pmela* alleles, and the available data are most consistent with a dominant phenotype caused by toxicity of the disrupted repeat domain (e.g., that disrupts normal formation of a functional amyloid to scaffold melanin deposition, Figure 6B); but we cannot rule out that reduced abundance of Pmela in the heterozygous fish may have exacerbated or caused the observed phenotypes.

Akin to the new ua5030 allele reported here, missense mutations in *PMEL* homologs across various species are most likely to cause dominant phenotypes with more severe outcomes, whereas null mutations show only the dilution of body pigmentation [17]. The hypothesis that dominant *PMEL* mutations are toxic has previously been explored and supported through comparisons of different *Pmel* genotypes in chickens [24,25]. An insertion mutation in the transmembrane domain causes a dominant white plumage. When a deletion is introduced earlier in this allele, the phenotype is tempered to an intermediate “smoky” colour (akin to a null phenotype). Remarkably, in a separate lineage, a different mutation in *Pmel* also makes a dilute “dun” colouration.

Further work on this mechanism is warranted, including an assessment of fibril structure that could potentially shed light on the toxicity of the different mutants. Variation in mutated fibrils could be assessed within depigmented melanosomes of fish similar to what has been carried out previously with PMEL in cell culture [1]. The repeat region of PMEL is O-glycosylated, and this could play an important role in tertiary and quaternary amyloid protein structures [2,3,26,27,28,29,30,31]. Furthermore, while the PMEL protein is highly conserved across vertebrates, the repeat domain sequence is built from different repetitive sequences between taxa, and this creates challenges in the modelling of the exact disease-causing mutations [17,18]. The factors responsible for the change in the functionality of the repetitive region could help explain the variable disease course in humans with *PMEL* mutations, in addition to contributing to our general understanding of the role of repetitive regions in amyloidogenic proteins. Some ideas include roles for O-glycosylation not only affecting tertiary and quaternary protein structures but also shielding the cell membranes from toxic PMEL amyloid, and/or facilitating an increased binding of toxic intermediates from pigment production that could otherwise cause cell stress [8]. Furthering the knowledge of amyloid biology concerning mechanisms that perturb protein aggregation, or cause amyloid-related toxicity, has the possibility of eventually aiding the amelioration of the course of amyloidogenic diseases.

Other factors (genetic background, effects on developmental rate and chance) may affect this phenotype, as there was phenotypic variation within genotypes. Photoreceptors were only in one of the *ua5022* mutants, and only one of the *ua5030* mutants had sufficient photoreceptors to view consistent abnormalities in outer segments. Phagocytosis and the blunting of photoreceptors were only observed in *fading vision* fish (Figure 6) [20]. This could be explained by a partial loss of function or a partial compensation mechanism (e.g., by the *pmelb* paralog) that causes variability in homozygous zebrafish *pmela* mutants (or alternatively other unexpected genetic anomalies). Larger sample sizes would be required to rule out any technical artefacts. A parallel to this within-genotype variance has been observed in other *Pmel* mutants, where the dominant white phenotype in chickens has decreased penetrance in males [24].

### 3.2. Use of Larval and Adult Zebrafish as Models of Pigmentary Glaucoma

It is important to be able to assess disease symptoms in a way that is relevant to what is observed in human patients to be able to best provide translational results. In diseases that variably progress, like pigmentary glaucoma, non-invasive, easily repeatable and longitudinal measurements are of great utility in tracking disease progression within individual research subjects. Intraocular pressure is part of the minimum database assessed routinely in ophthalmic patients and is an important risk factor and treatment target for glaucoma [23].

Rebound tonometry provides a relatively “tank-side” diagnostic tool to measure intraocular pressure in zebrafish glaucoma models in a minimally invasive way. Previously described methods in zebrafish, although considered the gold standard for accuracy, are not easily adopted or amenable to repeated measures [23,32].

Mouse would be a natural choice for a preclinical model of PMEL; however, no ocular phenotypes have been reported in *Pmel* mouse mutants. Indeed, despite multiple null mutations being discovered in different species, ocular phenotypes have only been reported in dog, horse and zebrafish [5,17,18].

Rebound tonometry did not show a difference in intraocular pressure between the zebrafish genotypes, which could mean that PMEL disruption is not the direct molecular cause of this glaucoma symptom. However, the data should be interpreted with caution, as the lack of a clear difference could be due to the variability and/or the later onset/recovery of phenotypes in fish (Figure 4A). To fully assess our new *ua5030* mutant, it would be ideal to serially measure it (and other genetic causes of heightened intraocular pressure in zebrafish) in concert with OCT until senescence. That approach might determine a timeline and elucidate further details of the pathobiological relationship between intraocular pressure and ganglion cell death beyond pigmentary glaucoma progression. However, we must also consider the limitations of a zebrafish model. The ocular anatomy of humans and zebrafish is sufficiently different that conformational/developmental changes that predispose to pigmentary glaucoma may not be fully represented in the zebrafish anatomy. In humans, the aqueous humour of the anterior segment is drained through the circumferential trabecular meshwork cells, and this can be blocked, heightening intraocular pressure, through either the clogging of the meshwork with debris or through damaging these specialized cells [33]. Zebrafish have a different structure to drain aqueous humour, the ventral canalicular network [33]. Additionally, retinal regeneration is robust in zebrafish, and this apparent resolution of the ocular phenotype could reflect their resilience and redundancy outshining the human ability to overcome ocular damage [34,35,36,37,38]. Our model is likely most relevant to the hypothetical cytotoxic pathomechanisms of pigmentary glaucoma (especially regarding the dystrophic pigment cells). However, the human mutations could have a unique toxic aspect to them resulting in pigment sloughing to the trabecular meshwork, or other unique pathologies within the eye. However, this may not be practical for animal disease modelling considering the large variation in the sequence of the PMEL repeat domains between animal clades [17].

The hypothesis that *PMEL* causes pigmentary glaucoma via anatomical changes in the anterior segment is somewhat supported by both zebrafish homozygous phenotypes and the *ua5030* heterozygote mutant phenotypes. All three genotypes showed a decreased eye size starting at 3 dpf that persisted until 7 dpf (Figure 3D), in addition to the previously described changes in the shape of the anterior segment of the *ua5022* homozygous larvae, all suggestive of Pmela affecting the anatomical development of the eye [1].

The differences between the larval and adult phenotypes could simply be reflective of ontogeny. However, it is worth mentioning that there was a much lower survival rate among the homozygous mutant larvae than among the wildtype larvae, which could be suggestive of only the mildly affected individuals surviving to adulthood (e.g., larvae with better vision would undoubtedly be more successful at feeding and surviving). Secondly, as we were originally interested in the repetitive domain, Pmela was the natural choice as a functional homolog to PMEL, as zebrafish Pmelb lacks an obvious repetitive domain and homologous trafficking cues [17]. However, it is possible that *pmelb* or other genes could compensate for some functions of *pmela* in adult fish.

### 3.3. Speculations about Dysamyloidosis and Potential Prion-like Pathomechanisms

Amyloidosis refers to the formation of toxic amyloid that is thought to be causal of infamous neurodegenerative diseases, including Alzheimer’s disease. Typically, the amyloidosis aetiology is accompanied by a prion-like mechanism that explains the spreading progression of the disease: seeds of disease-associated amyloid spread to new cells and tissues, where they nucleate the formation of more amyloid.

Functional amyloids, on the other hand, are rare in vertebrates but offer an opportunity to gain insight into these devastating diseases: e.g., how do cells manage the long-term abundance of amyloid [7,8,9,10,11,12]? Are the amyloid disease outcomes solely a toxic gained function or can they also be explained by disrupted protein function due to its misfolding and aggregation [7,8,9,10,11,12]? Amongst the few functional amyloids known, PMEL offers a truly unique opportunity to address these questions: when disrupted in patients, PMEL causes pigmentary glaucoma, which is a progressive neurodegenerative disease. Thus, clinically relevant PMEL mutations allow for insight into the aetiology when amyloid function is disrupted. We suggest that the term “*dysamyloidosis*” can best capture this pathomechanism, wherein functional amyloid is converted to dystrophic amyloid (via mutation, protein misfolding, prion-like spreading, etc.). While human pedigrees of pigmentary glaucoma show that mutations in PMEL’s repeat domain lead to dominant inheritance, until this work, no animal model was available to confirm this pattern or investigate its aetiology. Here, the first animal model with PMEL mutations that target the repeat domain was able to recapitulate a dominant inheritance in pigmentation defects and apparent amyloid loss of function.

It remains to be determined whether PMEL amyloid can act in a prion-like fashion, but our cell culture work (Figure 1, mimicking classic prion-like experiments with the transfer of supernatant to naïve cells) demonstrates the propensity of PMEL to spread to other cells and tissues in a non-cell-autonomous fashion. This includes the uptake of PMEL by trabecular meshwork cells, which are a focal point of pigmentary glaucoma (where dystrophy could cause glaucomatous increased IOP). Moreover, the dominant inheritance associated with PMEL in pigmentary glaucoma in patients, and in zebrafish and chicken pigmentation, suggests that when even a minority of the PMEL protein molecules are dystrophic that is sufficient to induce disease. Therefore, PMEL amyloid likely displays a toxic gain of function, and that may be mediated by inducing dysfunction in normal PMEL via intermolecular interactions (e.g., the amyloid is no longer able to function properly to scaffold melanin deposition). Further work is critically needed to investigate whether this speculative prion-like mechanism can be supported at the biochemical level, such as testing whether mutant PMEL can induce normal PMEL to alter its amyloid character and lose its normal functions.

### 3.4. Conclusions

Despite not perfectly recapitulating human pigmentary glaucoma, it is exciting to have developed a mutant that more closely mimics the genetic cause and inheritance of pigmentary glaucoma while displaying relevant pigmentary and ocular phenotypes. Further investigation into what occurs at the molecular level within these fish is warranted to determine the potential toxicity of mutations in the repeat domain and how they are responded to. This has translational implications for amyloid biology and treatment beyond pigmentary glaucoma.

## 4. Methods

### 4.1. Animal Care

Zebrafish were housed at the University of Alberta aquatics facility and used under the Animal Use Protocol 00000077 approved by the Biosciences Animal Care and Use Committee, which implements the Canadian Council for Animal Care guidelines. We generated a mutant allele of *pmela* in zebrafish (allele ua5030, see below) and also utilized a previously published allele *pmela*^ua5022^ ZFIN id ZDB-ALT-191119-1 [1]. All zebrafish were on the AB genetic background and maintained under standard conditions.

Zebrafish also express a second paralog, *pmelb*, and its gene products could play a role in this biology. However, the Pmelb protein is predicted to lack a repeat domain [17] and so is not considered further here.

### 4.2. CRISPR/Cas9 Targeted Mutagenesis of the Zebrafish Homolog of PMEL

Mutagenesis with CRISPR/Cas9 employed established methods [39] that closely resemble other recent mutagenesis methods in our group [40]. Oligos were purchased from Integrated DNA Technologies and used as per previous methods (ATTTAGGTGACACTATATGGAGCTTCGACTGCAGGTTGTTTTAGAGCTAGAAATAGCAAG, ATTTAGGTGACACTATAGGTTCGGCTTCAATAACTGCGTTTTAGAGCTAGAAATAGCAAG; and constant oligo: AAAAGCACCGACTCGGTGCCACTTTTTCAAGTTGATAACGGACTAGCCT TATTTTAACTTGCTATTTCTAGCTCTAAAAC) to generate gRNA against exon 7 of *pmela* on chromosome 11. Wildtype zebrafish embryos (AB strain) were injected with gRNA and Cas9 protein, and F1 progeny were monitored for pigment defects. Mutations in *pmela* were confirmed via sequencing. Briefly, flanking primers (fwd: GTGGTCGCAGTTTCTCCTCA; rev: GCAATGATTAGTGCGGCCTG) were used to amplify the gRNA-targeted region, and PCR products were TA-cloned into pGEM-T (Promega) vector. Sanger sequencing (University of Alberta, MBSU) revealed the mutant allele, designated as allele *ua5030*, characterized by a 12 bp in-frame deletion that would normally encode four residues at the C-terminal portion of the repeat domain.

### 4.3. Antibody Production

A custom polyclonal antibody was raised against the repetitive region of zebrafish Pmela by GenScipt USA, Inc. (Piscataway, NJ, USA). The antigen represented five Pmela repeats of about 22 residues each, spanning residue 382-511 of protein B3DJDP, cloned with a C-terminal His_6_ tag and expressed in *E. coli*. This antigen was delivered to rabbits, and anti-Pmela antibody was affinity-purified from their serum.

### 4.4. Transmission Electron Microscopy and Image Analysis

Whole embryos aged five days post-fertilization (dpf) were fixed in 2.5% glutaraldehyde and 2% paraformaldehyde in 0.1 M phosphate buffer (pH 7.2–7.4). They were washed in phosphate buffer prior to treatments with 1% osmium tetroxide. They were serially dehydrated using ethanol and then cured in Spurr resin. A Reichert–Jung Ultracut E Ultramicrotome was used to cut sections to a 70–90 nm thickness prior to staining them with uranyl acetate followed by lead citrate on a grid. A Philips FEI Margagni 268 transmission electron microscope operating at 80 kV was used with a Gatan Orius CCD camera to visualize the samples.

One representative image was taken from the limits of the retinal pigmented epithelium and two from the mid-retinal area for each fish. Fiji win 64 was used to measure the Feret’s diameter (longest straight line within a shape), and the grayscale standard deviation (each pixel was assigned a numeric value representative of how light or dark it was, allowing the average and standard deviation to be calculated) within each melanosome. The measurements were all averaged to a singular value for each fish prior to conducting ANOVAs with post hoc Tukey tests using Graph Pad Prism 9.3.1.

### 4.5. Optical Coherence Tomography

Optical coherence tomography (OCT) was conducted as per recent works [40,41] using a mounted Enyisu R-Series OCT (Bioptgen Inc., Durham, NC, USA). Fish were anaesthetized and positioned for imaging, and retinal images were captured using InVivoVuee 2.4 OCT management software (Bioptigen Inc., Durham, NC, USA) at the location of the optic nerve. A measure of the full thickness of the retina and the proportion of it that each layer occupied was taken from both sides of the optic nerve as soon as the layers were clearly identifiable using Fiji. A Mann–Whitney test was performed, comparing each genotype with an age-matched control.

### 4.6. Intraocular Pressure in Adult Zebrafish by Rebound Tonometry

Individual zebrafish were anaesthetized via immersion in tricane methanesulfonate, (DIN 02168510; Syncaine, Syndel, Nanaimo, BC, Canada) and facility water until operculation was slowed/irregular. They were propped up to be in the anatomical position at the edge of the counter. The tonometer (Icare Tonovet plus, TV011, © 2023 Icare Finland Oy, Vantaa, Finland) needs to stay upright during use, and this allowed space for it below the height of the counter. The Tonovet plus was kept level and pointed at the middle of the globe prior to propelling the probe. The Tonovet plus requires 6 measurements, and it discards the highest and the lowest prior to averaging the remaining 4. It assigns a colour score for variance. It discards any measurements where the probe is too far or too near and gives correction to the user. Only green (the most consistent) measurements were accepted, and two to three (averaged) measurements were averaged for each eye. The right eye was always measured first. If the eyes were becoming dry (probe sticking as opposed to rebounding), a drop of the anaesthetic/water was put over the head. The two eyes were then averaged, and a Kruskal–Wallis test was used to compare between genotypes using Graph Pad Prism 9.3.1.

### 4.7. Western Blots

Zebrafish larvae were euthanized and added to 1 mL de-yolking buffer (filter sterilized mixture of 2.75 mL 1 M NaCL, 90 microlitre 1 M KCl, 62.5 microlitre 1 M NaHCO_3_, 47.1 mL distilled H_2_O) with a protease inhibitor. They were thoroughly mixed with a pipette and vortexed prior to being centrifuged. The pellet was resuspended in fresh wash buffer (5.5 mL 1 M NaCL, 175 microlitre 1 M KCL, 500 microlitre Tris pH 8.5, 135 microlitre CaCl_2_, 44.2 mL distilled H_2_O) and centrifuged. Cell lysis buffer (4 mL 0.5 M Hepes, 20 mL 100% glycerol, 10 mL 5 M NaCl, 0.039 g MgCl_2_, 40 microlitre 0.5 M EDTA, 100 microlitre Triton X-100) was added to either pelleted larvae or whole adult eyes prior to being homogenized and centrifuged. The supernatant was collected for analysis.

NuPAGE LDS sample buffer and denaturing additive (Thermofisher Scientific, Waltham, MA, USA) were added to all samples prior to loading on NuPage Bis-Tris 4-12% gels (Thermofisher Scientific). Gels were run at 150 V for 2 h and transferred to PVDF membranes overnight at 30 V. Blots were blocked in Intercept Blocking Buffer (Licor, 927-70001) and incubated with a custom Pmela primary antibody (Rabbit anti-Pmela-001 (1:50), GenScript, described above) and anti-actin as a loading control (Rabbit anti-actin (1:1000), Sigma-Aldrich, A2066, St. Louis, MO, USA). Blots were washed for 5 min 3 times in PBS-T (0.1%) and incubated with IRDye Goat anti-Rabbit 800 CW fluorescent secondary antibody for 1 h at room temperature. Blots were scanned and analyzed on an iBright Scanner (Thermofisher Scientific).

### 4.8. RT-qPCR

RNA was extracted from larvae using an RNeasy Mini Kit (Qiagen, 74104). RNA was quantified, and cDNA was created using qScript Tm cDNA SuperMix (Quanta Bio 95048-500). qPCR was run using a SYBR green master mix (sourced in-house from the Molecular Biology Service Unit at University of Alberta) and 7500 Fast Real-Time PCR System. Primers were previously validated to MIQE standards [1] and used to amplify *pmela* and two reference genes, *actb* (β-actin) and *rpl13a*. Relative expression was calculated as follows: 2^ΔCt GOI^/2^ΔCt Ctrl^. Gene expression was calculated for both reference genes, and the geometric mean of the two values taken. Significance was determined using a One-Way ANOVA with Dunnet’s Correction for Multiple Testing in GraphPad Prism (v9.5.1).

### 4.9. CRISPR-Cas9 Editing of Melanoma Cells

The melanoma cell line SK-MEL-5 (ATCC #HTB-70) was a kind gift from Dr. Alan Underhill (University of Alberta), and it was cultured in high-glucose Dulbecco’s Modified Eagle’s Medium (DMEM) (ThermoFisher #11995) containing 10% Fetal Bovine Serum (FBS) (Sigma #F1051) plus 1% Penicillin/Streptomycin (ThermoFisher #15140). Knockout of the PMEL gene in SK-MEL-5 cells was achieved using the 2-part guideRNA option of the Alt-R CRISPR-Cas9 System and predesigned crRNA reagents from Integrated DNA Technologies (#Hs.Cas9.PMEL.1.AA & Hs.Cas9.PMEL.1.AB). Successfully transfected cells were FACS-sorted (owing to ATTO550-labelled tracrRNA), and clones were cultured separately. Knockout of PMEL expression was confirmed via Western blotting [1] with the HMB45 antibody (Novus Biologicals #NBP2-44520) (detects Mα and MαC bands) and via a sequence analysis of individual PCR-cloned alleles. The loading control antibody was for alpha Tubulin (DM1A, Santa Cruz Biotechnology #sc-32293). The CRISPR clone SK5PA04 carries compound heterozygous frameshift mutations: c.403_410del,c.412_570inv [p.Gly135fsMet*27] and c.391_411del,c.412_570inv [p.Ile131fsMet*27], relative to Genbank accession NM_001200054.1.

### 4.10. In Vitro Analyses of Human PMEL Variants

The protocols for plasmid cloning, transfection, Western blotting and immunocytochemistry were previously described [1]. SK5PA04 cells were transfected with Lipofectamine 2000 (ThermoFisher #11668) as per the manufacturer’s protocol using the cell culture conditions described in the reference above. Cells were lysed (with 0.1% sodium dodecyl sulfate, 0.5% sodium deoxycholate, and 1% IGEPAL CA-630 in phosphate-buffered saline (1x PBS)), and 25 μg of lysates was blotted to nitrocellulose. V5-tagged expression constructs were detected with Rabbit anti-V5 antibody (Sigma #V8137) (detects P1 and Mβ bands) and visualized using chemiluminescence (SuperSignal West Femto Maximum Sensitivity Substrate, ThermoFisher #34096). Conditioned media (CM) were also collected from transfected SK5PA04 cells (1 day post-transfection, growth media from 10 cm plates were replaced with 5 mL OptiMEM media (ThermoFisher #31985) and conditioned for one day before collection). Aliquots of 1 µL CM were applied to cultures of TM-1 cells, a transformed human trabecular meshwork cell line [42], which was a gift from Dr. Vincent Raymond (Université Laval) and was cultured in low-glucose DMEM (ThermoFisher #11885) containing 10% FBS plus 1% Penicillin/Streptomycin. TM-1 cells were grown on coverslips in 6-well dishes and incubated with CM for 48 h. TM-1 cell viability was assessed with a LIVE/DEAD Viability/Cytotoxicity Kit for mammalian cells (ThermoFisher #L3224). Cells were fixed with 4% paraformaldehyde, permeabilized with 0.1% NP-40, blocked with 6% normal donkey serum + 0.5% BSA in 1x PBS and then incubated with the primary antibodies (Rabbit anti-V5 at 1:500 and Mouse anti-HMB45 at 1:100) for 1 h. Fluorescent secondary antibodies (Donkey anti-Rabbit-Alexa488 and Donkey anti-Mouse-Alexa647, Jackson Immuno Research Laboratories, Inc., Westgrove, PA, USA) were incubated at 1:500 dilution for 1 h. Nuclei were visualized via staining with Hoechst dye (1 μg/mL), and coverslips were mounted with Fluoromount-G (ThermoFisher #00-4958-02). Imaging was performed with an Axio Imager M2 fluorescence microscope (Carl Zeiss Canada Ltd., North York, ON, Canada).

### 4.11. Bioinformatics

Geneious Prime 2020.1.2., created by Biomatters (Boston, MA, USA), was used to create the protein repeat alignment figures. Refer to Chrystal et al. for how repeats were originally located and defined [17].

## Data Availability

All available data is presented in the manuscript.

## Supplementary Materials

The following supporting information can be downloaded at https://www.mdpi.com/article/10.3390/ijms241914423/s1.
