# Supplementary material for: Disrupting the Repeat Domain of Premelanosome Protein (PMEL) Produces Dysamyloidosis and Dystrophic Ocular Pigment Reflective of Pigmentary Glaucoma
Source: Int J Mol Sci. 2023 Sep 22;24(19):14423. doi: 10.3390/ijms241914423 (PMC10572516; doi:10.3390/ijms241914423)
Supplement: Supplementary file 1 [file ijms-24-14423-s001.zip › ijms-2573956-supplementary.pdf]

Supplemental Figures

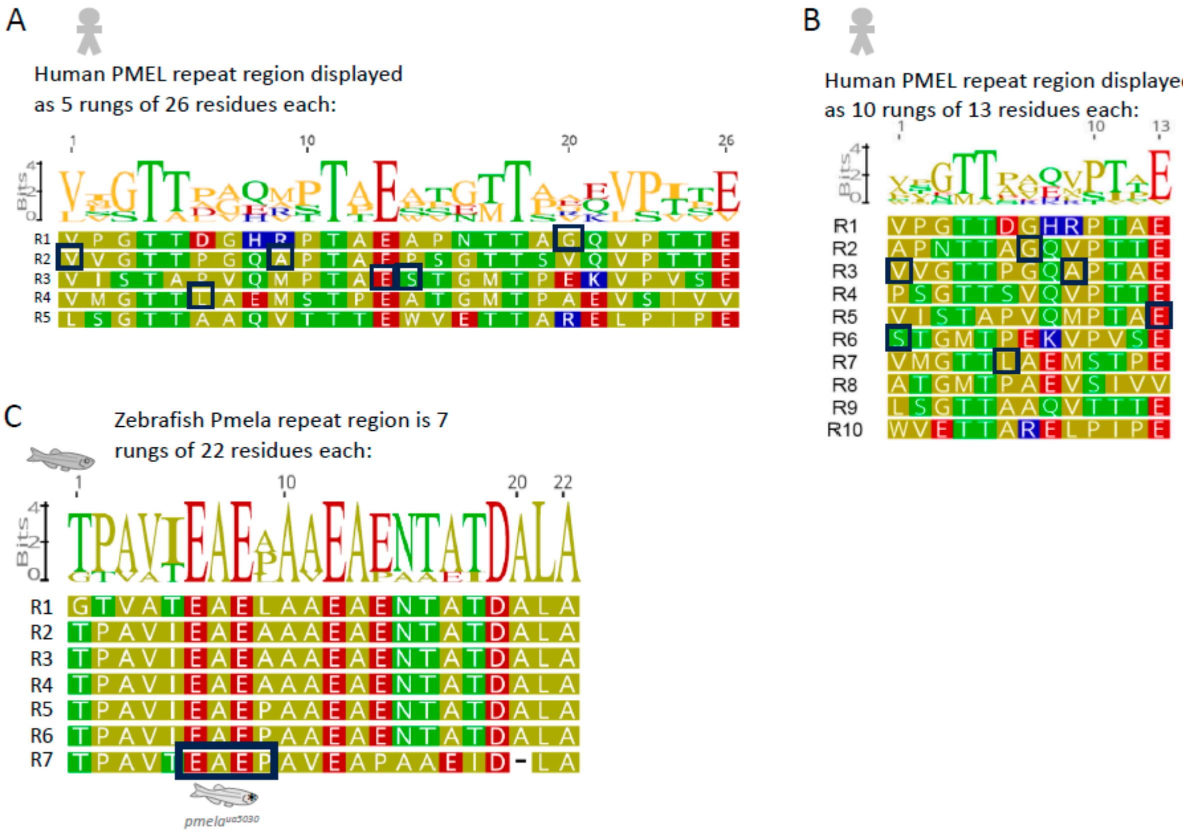

Figure S1. Human and zebrafish homologs of PMEL share a homologous repeat region but differ in their details. This figure relates to Figures 1 and 2, and it shows the complexity in residue identity that was omitted there to help communicate the repetitive aspects. Human and zebrafish repeat domains both have rigidly repeating sequences of amino acids; the content and size of the repeats vary between species, but the repeats are rigidly conserved to each other within each species. **A, B.** Human PMEL repeats have typically been schematized as 10 repeats of 13 residues (B), but it is equally valid to consider them as 5 repeats of 26 residues (A). Boxes indicate patient variants that cause pigmentary glaucoma. **C.** Zebrafish Pmela repeat domain; same data as in Fig 2F, reformatted here for ease of comparison with repeats of the human sequence. Boxed area indicates four residues deleted in allele ua5030.

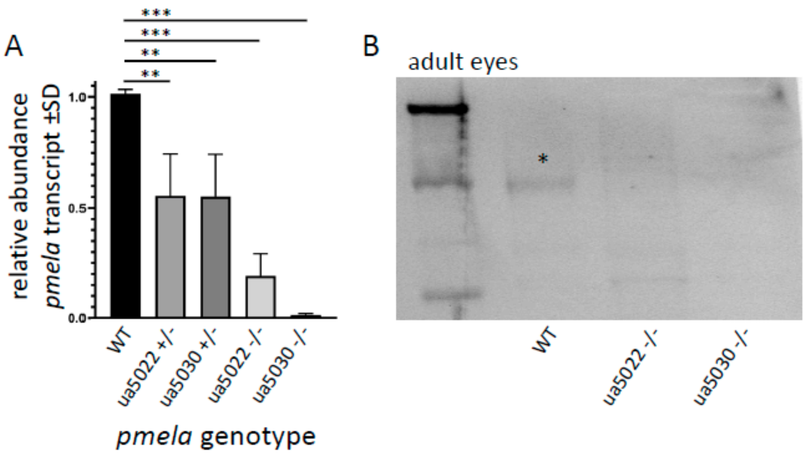

C

Primer Name	Sequence
pmela qPCR Forward	GCGACACTCGGAGTTCTGTT
pmela qPCR Reverse	TCACACCACGCGTCCCAGCA
rpl13a qPCR Forward	TCTGGAGGACTGTAAGAGGTATGC
rpl13a qPCR Reverse	AGACGCACAATCTTGAGAGCAG
actb qPCR Forward	GGGACAGGTCATCACCATTG
actb qPCR Reverse	GATGTCGACGTCACACTTCA

Figure S2. Efficacy of targeted mutagenesis for disrupting *Pmela* gene products. This figure relates to Figure 2D,E. **A.** *pmela* transcript abundance is reduced in both alleles of *pmela* mutants, presumably via decay of mutant transcript. N=3 biological replicates per genotype, each replicate composed of five larvae. **p<0.01, ***p<0.001. **B.** Adult zebrafish eyes (3 years old) contain Pmela protein in wildtype (WT) zebrafish, but Pmela is not detectable in homozygous mutants from either allele. Blot was probed with a custom anti-Pmela antibody raised against the repeat region. **C.** Primers used for RT-qPCR.

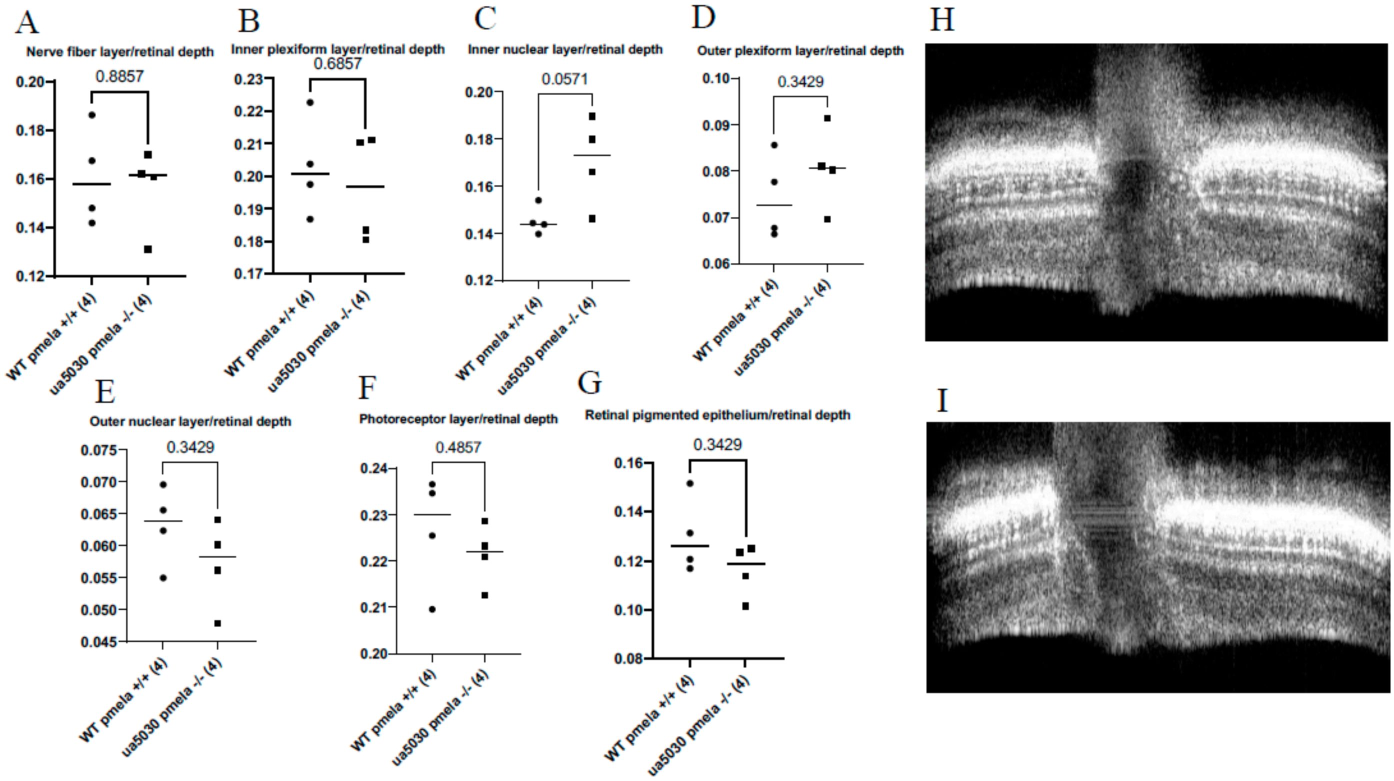

Figure S3. Adult homozygous *pmela* ^{ua5030} individuals (panel H) are not measurably different from wildtype individuals (panel I) using OCT. Accompanies Figure 4.

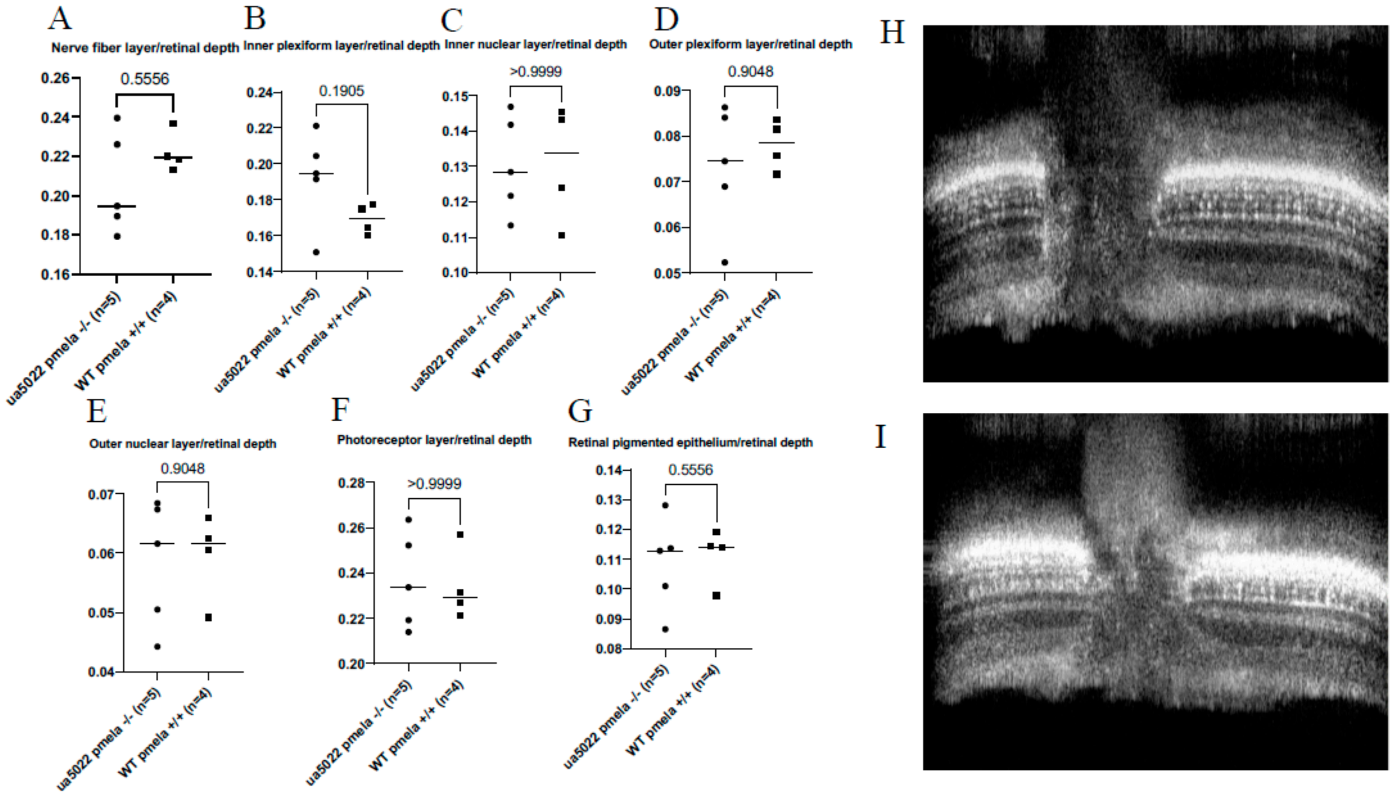

Figure S4. Adult homozygous *pmela* ^{ua5022} individuals (panel H) are not measurably different from wildtype individuals (panel I) using OCT. Accompanies Figure 4.

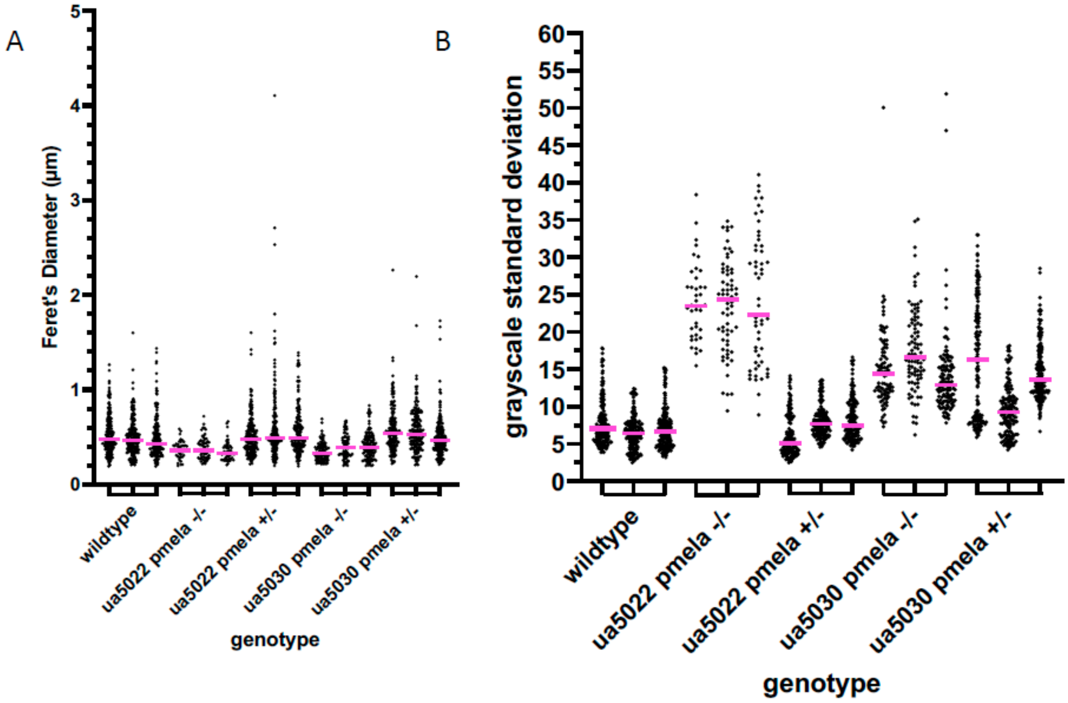

Figure S5. Variation in phenotypes in each melanosome is considerable within each individual animal, and this variation is amplified in *pmela* mutant genotypes. Accompanies Figure 5C,D, which compares the means of these values when considering each fish as the statistical unit. Data from three fish per genotype are displayed. A. Variation in size of each melanosome within each individual animal is substantial. B. Variation in pigmentation *within each melanosome* is higher in *pmela* mutant genotypes. Notice the large variation within all individuals heterozygous for *pmela*$^{+/ua5030}$.
